# Comparison of Aerobic Fitness in Patients with Schizophrenia and Healthy Adults

**DOI:** 10.1192/j.eurpsy.2026.10540

**Published:** 2026-07-31

**Authors:** A. H. Bayraktar Özdemir, E. T. Özel Kızıl, G. Ömercioğlu, A. Demirci Şahin, Z. E. Erden, D. Onar, Z. Uçar Hasanlı, M. Baştuğ

**Affiliations:** 1 Department of Psychiatry; 2 Department of Physiology, Ankara University, Ankara; 3 Department of Psychiatry, Bakirkoy Training and Research Hospital for Psychiatry, Neurology and Neurosurgery, İstanbul; 4 Department of Psychiatry, Ankara Training and Research Hospital, Ankara, Türkiye

## Abstract

**Introduction:**

Individuals with schizophrenia face approximately 2.5 times higher all-cause mortality and nearly twice the risk of cardiovascular disease(CVD)-related mortality compared to the general population (Correll et al. World Psychiatry 2022; 21:248–271).Aerobic fitness is defined as the ability of the circulatory and respiratory systems to supply oxygen to skeletal muscles during sustained activity (Franklin et al. Am J Prev Cardiol 2022; 12:100424).Low aerobic fitness is a strong, independent predictor of both CVD and mortality, with prognostic value comparable to diabetes and other established risk factors(Kodama et al. JAMA 2009; 301:2024–2035).In schizophrenia, poor aerobic fitness is also associated with clinical outcomes such as increased illness severity, particularly negative symptoms, and decreased global functioning(Vancampfort et al. J Ment Health 2015).Improving aerobic fitness thus represents a key target for reducing cardiometabolic risk and illness burden.

**Objectives:**

Given the limited and inconclusive data, this study aims to compare aerobic fitness between individuals with schizophrenia and healthy controls to determine whether patients with schizophrenia are at greater cardiovascular risk, and to explore its relationship with illness severity.

**Methods:**

The study included 58 participants:29 individuals with schizophrenia and 29 healthy controls.All participants underwent a cardiopulmonary exercise test (CPET) using a cycle ergometer to assess aerobic capacity.Maximal oxygen consumption (VO₂max) was measured via respiratory gas analysis.For participants who did not reach VO₂max, the highest achieved value (VO₂peak) was recorded, and VO₂peak/max/kg was used to define exercise capacity.Symptom severity in patients was assessed using the Positive and Negative Syndrome Scale (PANSS).Group differences in exercise capacity were analyzed using the Mann–Whitney U test, and correlations between PANSS scores and aerobic capacity were examined by Spearman’s test.

**Results:**

Aerobic fitness (VO₂peak/kg) was significantly lower in the schizophrenia group compared to healthy controls(p= .001). No statistically significant correlation was found between VO₂peak/kg values and total PANSS scores,which reflect illness severity, in the schizophrenia group(p= .666). Similarly, no significant associations were observed between VO₂peak/kg and the PANSS subscales, including positive symptoms (p= .089), negative symptoms (p= .623),or general psychopathology scores(p= .962).

**Conclusions:**

Individuals with schizophrenia demonstrated significantly lower aerobic fitness than healthy adults, confirming a substantial cardiorespiratory fitness deficit in this population. While no significant association was observed between aerobic fitness and symptom severity, this may reflect the possibility that these domains are not directly related in clinically stable patients.

**Disclosure of Interest:**

None Declared

